# The Spatial Organization of Proton and Lactate Transport in a Rat Brain Tumor

**DOI:** 10.1371/journal.pone.0017416

**Published:** 2011-02-24

**Authors:** Emmanuelle Grillon, Régine Farion, Katell Fablet, Michel De Waard, Chung Ming Tse, Mark Donowitz, Chantal Rémy, Jonathan A. Coles

**Affiliations:** 1 Unit 836, Institut National de la Santé et de la Recherche Médicale, La Tronche, Isère, France; 2 Grenoble Institut des Neurosciences, Université Joseph Fourier, Grenoble, France; 3 Gastroenterology Division, Departments of Physiology and Medicine, Johns Hopkins University School of Medicine, Baltimore, Maryland, United States of America; 4 Centre for Biophotonics, University of Strathclyde, Glasgow, United Kingdom; City of Hope National Medical Center and Beckman Research Institute, United States of America

## Abstract

Tumors create a heterogeneous acidic microenvironment which assists their growth and which must be taken into account in the design of drugs and their delivery. In addition, the acidic extracellular pH (pHe) is itself exploited in several experimental techniques for drug delivery. The way the acidity is created is not clear. We report here the spatial organization of key proton-handling proteins in C6 gliomas in rat brain. The mean profiles across the tumor rim of the Na^+^/H^+^ exchanger NHE1, and the lactate-H^+^ cotransporter MCT1, both showed peaks. NHE1, which is important for extension and migration of cells *in vitro*, showed a peak 1.55 times higher than in extratumoural tissue at 0.33 mm from the edge. MCT1 had a broader peak, further into the tumor (maximum 1.76 fold at 1.0 mm from the edge). In contrast, MCT4 and the carbonic anhydrase CAIX, which are associated with hypoxia, were not significantly upregulated in the rim. The spatial distribution of MCT4 was highly correlated with that of CAIX, suggesting that their expression is regulated by the same factors. Since protons extruded by NHE1 diffuse away through extracellular clefts, NHE1 requires a continuous source of intracellular protons. From the stoichiometries of metabolic pathways that produce or consume H^+^, and the greater availability of glucose compared to oxygen in most parts of a tumor, we support the classic view that most of the net proton efflux from C6 gliomas originates in glycolytic formation of lactate and H^+^ inside the tumor, but add that some lactate is taken up into cells in the rim on MCT1, and some lactate diffuses away, leaving its associated protons available to re-enter cells for extrusion on NHE1. Therapeutic inhibition of NHE1, MCT1 or CAIX is predicted to affect different parts of a tumor.

## Introduction

For a systemically administered drug to act preferentially on a tumor, the drug must recognize some specific characteristic of the tumor. The most widely-exploited characteristic is the rapid replication of DNA, which suffers from being shared by cells of the bone marrow, gut and hair roots. Inhibition of angiogenesis has given disappointing results in the long term [1]. Another specific characteristic of tumors, which concerns us here, is their unusually acidic extracellular pH (pHe). Most normal cells have an intracellular pH (pHi) of 7.1–7.2 and are bathed by extracellular fluid with a lower H^+^ activity corresponding to a pH of 7.4. In tumor cells, the transmembrane gradient of H^+^ activity is reversed: pHi can be greater than 7.3 [2]–[5], and pHe is typically in the range 6.4–7.0 [6]–[12]. The acidic pHe contributes to the invasiveness of tumors [2], [10], [11], [13], [14] and clinical trials are in progress using a pro-drug that is cleaved in the acidic environment to release an inhibitor of proton pumps [11]. Tumors overexpress extracellular proteinases [15], and these too are being used, in animal models, to target molecules by cleaving linkers and activating cell penetrating peptides [16]. Available data suggest that both the secretion and the catalytic activity of proteinases, including matrix metalloproteinases (MMPs), are increased at acidic pHs [17], [18] and that they promote the progression of tumors [19].

In addition to the unusual absolute value of pHe in tumors, the unusual transmembrane pH gradient can also be exploited to target drugs. Most simply, the pH gradient causes intracellular accumulation of drugs that are weak acids, while weak bases tend to be excluded (although the actual distribution may be dominated by other processes, such as extrusion of the drug on a drug transporter) [20]–[22]. More sophisticated use of the pH gradient includes attaching a drug or a fluorescent marker to a pH-sensitive carrier [23]–[25]. A different use that has been suggested for the acidic pHe is to image it non-invasively by magnetic resonance techniques as an aid to diagnosis or for following the effects of therapy [8], [26].

In order to optimize therapeutic strategies that exploit the acidic pHe, or target the mechanisms underlying it, it would be useful to understand what causes it. Extracellular H^+^ ions can diffuse freely through extracellular clefts and so the accumulation of H^+^ represented by an acidic pHe can only be maintained if H^+^ ions (or some other acid equivalent) are continually generated within, and exported from, the tumor cells [10], [27], [28]. The main source is the conversion of glucose to equal numbers of lactate-ions and H^+^ ions: C_6_H_12_O_6_ = 2C_3_H_5_O_3_^−^ + 2H^+^ [29]–[31]. In the steady state, lactate- must leave the cell at a rate equal to its production. Driven by their concentration gradient, lactate-ions leave the cells in one-to-one association with H^+^ ions, either by diffusion of neutral lactic acid, or on a cotransporter of the MCT class [31], [32]. The isoforms MCT1 (SLC16A1) and MCT4 (SLC16A3) are upregulated in at least some tumors [33], [34]. In the steady state, the concentrations of products of ancillary reactions, such as conversion of NAD to NADH, are recycled so they do not contribute to any net flux of acid equivalents. Hence, production of lactic acid decreases pHe without (in the steady state) acid-loading the cells. Despite producing large amounts of lactate, even in the presence of oxygen [29], [30], [35]–[37] parts of tumors also oxidize glucose completely to CO_2_ [5], [30]. In the steady state, all the CO_2_ generated must effectively leave the cell, and it can do this by diffusion through the lipid membrane [38],[39] or perhaps through aquaporins [40]. On arrival in the extracellular space, CO_2_ reacts with water in the presence of carbonic anhydrase (CO_2_ +H_2_O = H^+^ + HCO_3_^−^) and thereby makes a contribution to extracellular acidity [5]. CO_2_ is also in equilibrium with H^+^ and HCO_3_^−^ within the cell, so if HCO_3_^−^ were to continually leave the cell, oxidative metabolism would tend to decrease pHi. However, HCO_3_^−^ is generally transported into, rather than out of, cells [41], [42] and removal of CO_2_/HCO_3_^−^ increases pHi in U118 glioma cells [2]. The synthesis from glucose and glutamine of molecules for cell growth appears in most cases to consume H^+^ (see Discussion). Thus the net effect of metabolism, in conjunction with export of its products, appears not to decrease pHi in the steady state, but it does decrease pHe.

However, the unusual inward gradient of [H^+^] into tumor cells subjects them to an abnormally large influx of acid equivalents that leak through ion channels or are carried on imperfectly specific transporters. It is therefore unsurprising that mechanisms for exporting acid equivalents are upregulated in tumors. Upregulation has been reported of the Na^+^/H^+^ exchanger NHE1 (SLC9A12, ref. [3]), and of the plasma membrane V-H^+^-ATPase [43]. NHE1 is interesting because numerous studies have shown that in cancer cells growing or migrating *in vitro* it is concentrated at the leading edge of "invadopodia" [3], [44]–[47]. NHE1, which is the primary regulator of pHi in almost all normal cells, uses the inward electrochemical gradient of Na^+^ to extrude protons. Its greater activity at the leading edge of migrating cells causes a local increase in pHi and a decrease in pHe, both of which promote cell extension [46]. Raised pHi remodels the cytoskeleton, while lowered pHe modifies attachment to substrate and disrupts extracellular matrix [46], [48]. In addition to modifying pH, the NHE1 molecule contributes to cell migration by interacting directly with other macromolecules [47], [49]. Inhibition of NHE1 slows tumor growth [3], [50], [51]. These results suggest that in tumors *in vivo*, NHE1 may have a role beyond regulation of pHi and might be concentrated near the growing borders. As part of the present work, we tested this hypothesis for the case in a rat model of glioma, grown from the C6 cell line.

If NHE1 is extruding protons and creating a locally acidic pHe, then where do these protons come from? (They cannot simply re-enter the cell close to where they are extruded as this would destroy the acidic pHe.) Sonveaux et al [14] provide a hint: they showed that in SiHa tumors, MCT1 is highly expressed in the rim of the tumor, where the energy metabolism is likely to be oxidative. They suggest that here MCT1, instead of exporting lactate, takes it up, as a substrate for oxidative metabolism. They do not discuss proton fluxes, but protons will presumably accompany lactate. Although, as we shall show in the Discussion, these protons do not constitute the internal source for NHE1, Sonveaux et al [14] do introduce the idea of fluxes between tumor cells. We have therefore compared the spatial distribution of MCT1 in C6 rat gliomas to that of NHE1.

To obtain a fuller picture, we also looked at two other proteins associated with H^+^ transport. These are the MCT isoform, MCT4 (SLC16A3), which is present on astrocytes [52], can be induced by hypoxia via the Hypoxia-Inducible Factor 1α (HIF-1α [34]), and is upregulated in parts of some tumors [53], [54]. We have also looked at an isoform of the enzyme carbonic anhydrase, CAIX, which is also upregulated *in vitro* by HIF-1α and, like MCT4, has been found in hypoxic parts of tumors [55]–[60]. Carbonic anhydrases, which catalyse the reaction H^+^ + HCO_3_^−^ = CO_2_ + H_2_O, can facilitate the diffusion of proton equivalents through extracellular space [5], [61]. The presence of a carbonic anhydrase close to an H^+^ transporter can increase the transporter's efficacy [62].

Our results show that gene expression in the growing border of a C6 glioma is spatially organized and confirm the hypothesis that expression of NHE1 is upregulated in the growing rim of a tumor *in vivo*. We also also introduce a new technique for identifying pairs of proteins whose expression is regulated by common factors. Taken together with stoichiometric constraints, and the knowledge that low pHe favors tumor growth, the results suggest that lactate and H^+^ ions flow between cells in a way that is a compromise between efficient use of oxygen and glucose for cell growth, and the creation of localized pH microenvironments.

## Results

### NHE1 and MCT1 peak in the tumor rim

**Figure 1:**
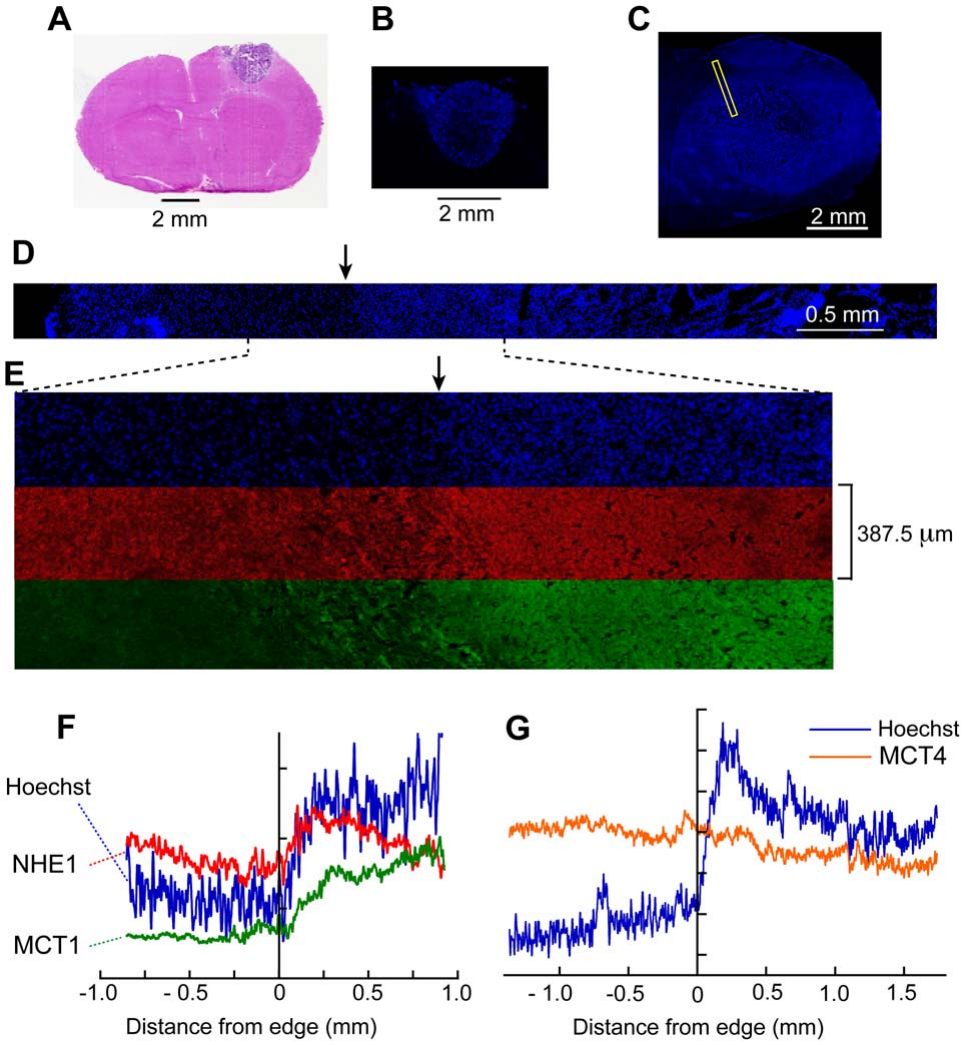
Immunofluorescence profiles across the tumor border. (A) A section of a small C6 glioma stained with haemotoxylin-eosin; (B) part of an adjacent section with nucleic acids stained with Hoechst 33342. (C) Another glioma stained with Hoechst and showing a strip crossing the border that was tilescanned. (D) The Hoechst tilescan of the strip indicated in (C). (E) Portions of the tilescan for Hoechst (blue) NHE1 (red) and MCT1 (green). (F) Intensity profiles of the strips in (E) with positive distances directed into the glioma. From the Hoechst profile, it was possible to identify the approximate position of the tumor border on the micrographs (arrows in D and E). (G) Intensity profiles from another section labeled for MCT4 showing it does not increase markedly in the rim.

**Figure 2:**
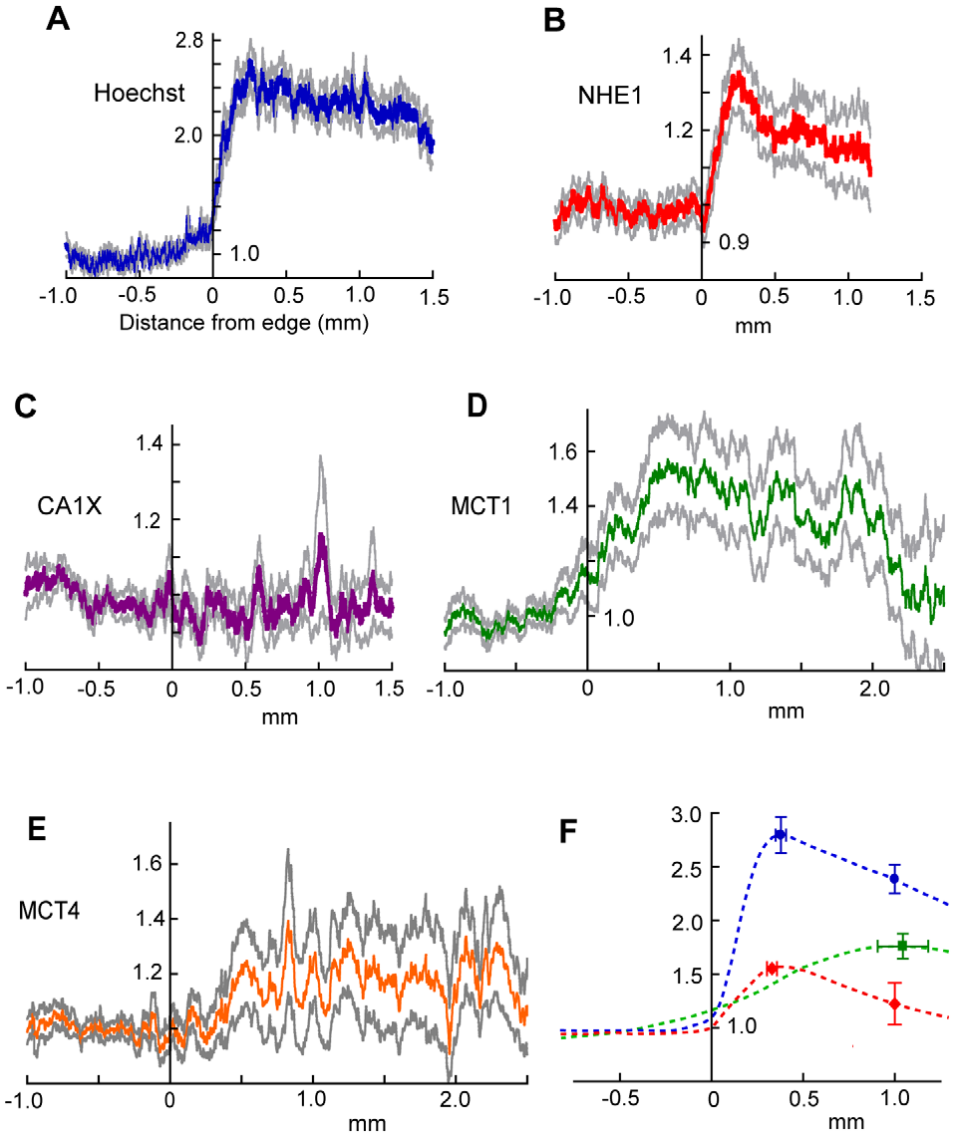
Organization of NHE1 and MCT1 in the tumor rim. (A – E). Labeling intensity (relative to outside the glioma) for Hoechst (average of 27 strips), NHE1 (22), CAIX (22), MCT1 (18) and MCT4 (16). Gray lines are an indication of SEM (see Methods). Note that the ordinate scales are not all the same. (F) Mean relative intensities ± SEM for peaks (Hoechst, NHE1 and MCT1) and values at 1 mm for Hoechst and NHE1. Schematic curves have been sketched in. The mean values were calculated from measurements on individual profiles and therefore do not correspond exactly with the averaged profiles in (A,B,D).

Coronal brain sections were selected that passed approximately through the equator of the C6 gliomas; the shorter diameters ranged from 1.94 to 8.0 mm (Fig. 1 A–C). In general, on each section, we stained nucleic acids with Hoechst 33342 and immunolabeled two of four proteins involved in proton transport: NHE1, MCT1, MCT4 and CAIX. The mean labeling intensities of large regions of interest (ROIs) within each labeled glioma were compared to the mean intensities in extratumoral tissue. The ratios were as follows. NHE1: mean  = 1.15, s.e.m.  = 0.10, n = 10 tumors, P for difference from 1 = 0.15. MCT1: 1.392±0.078, n =  10, P = 0.0007. MCT4: 1.34±0.18 n = 8, P = 0.105. CAIX: 1.14±0.14 n = 7, P = 0.36. Note that only for MCT1 was the ratio significantly greater than 1. All four proteins were detected in both normal brain and in the gliomas. Increased Hoechst labeling near the perimeters of the gliomas was evident (Figs 1B,C). For none of the four proteins was a convincing pattern of labeling apparent on visual inspection of the tumor rim, but patterns became evident on measured intensity profiles. In each section, we selected 1–3 sites on the tumor border where the Hoechst staining indicated a well-defined rim confronting neuronal tissue (rather than the brain surface). We imaged rows of 10–20 microscope fields to give composite images of bands of tissue each 387.5 µm wide with their long axes perpendicular to the border. The "tile scan" corresponding to the narrow rectangle in Fig. 1C is shown in Fig. 1D for the Hoechst fluorescence, and the part of this covering the glioma rim is expanded in Fig. 1E together with the corresponding images for NHE1 and MCT1. The intensity profiles along the images for each band were measured. We defined the edge of the tumor to be where the rise in Hoechst staining began (Figs. 1F,G, 2A). In almost all cases, NHE1 immunofluorescence showed a peak 0.25 – 0.4 mm into the tumor (Fig. 1F). We obtained 22 NHE1 profiles from 10 tumors, normalized each one so that the average intensity over 1 mm outside the tumor (−1 mm<x<0) was set at 1 and averaged them. No profiles were excluded from analysis.

In the mean NHE1 profile (Fig. 2B), the peak is well-defined. In contrast, the mean intensity of CAIX labeling showed no significant change across the rim (Fig. 2C). This absence of a peak in CAIX labeling suggests that the NHE1 peak was not an artifact, due, for example, to uneven tissue shrinkage. (In agreement with reports on other types of tumor, more intense CAIX labeling was present in small areas deep in the tumor, in tissue that was probably hypoxic [see 63]) The distance from the tumor edge of the NHE1 peak varied somewhat between individual profiles, so when the profiles were averaged (to give Fig 2B) the peak was somewhat flattened out. To avoid this, we also measured the peak amplitude and its position on the individual profiles, obtained average values for each tumor and calculated the overall means. Calculated in this way, the mean intensity of the NHE1 peak was 1.55 times the intensity outside the tumor (SEM = 0.10, n = 10 tumors, P = 0.0005). This figure still slightly underestimates the true difference in the expression of NHE1 protein between the peak and extratumoral tissue because no correction was made for non-specific labeling by the secondary antibody (see Supporting Information, Text S1, Figure S1). The anti-NHE1 peak was on average 0.330±0.027 mm from the edge. This position was not significantly different from the mean position of the Hoechst peak (0.379±0.029 mm).

Labeling of MCT1, which can transport lactate and H^+^ either out of or into cells [64], peaked near the rim (Fig. 2D); the average distance of the MCT1 peak from the edge was 1.05±0.14 mm (9 gliomas) significantly greater than the distance of the NHE1 peak (P = 0.0001). Mean values for the peaks and their distances from the tumor edge for Hoechst, NHE1 and MCT1 are shown in Fig. 2F. For MCT4, as for CAIX, we did not detect a significant increase in labeling in the rims of these gliomas (Fig 2E). The means of the ratios of the intensity at 1 mm from the edge to the intensity outside were, for MCT4, 1.31, S.D. = 0.47, n = 8 and, for CAIX, 1.17, S.D. = 0.35, n = 7.

### MCT4 and CAIX are spatially correlated

**Figure 3:**
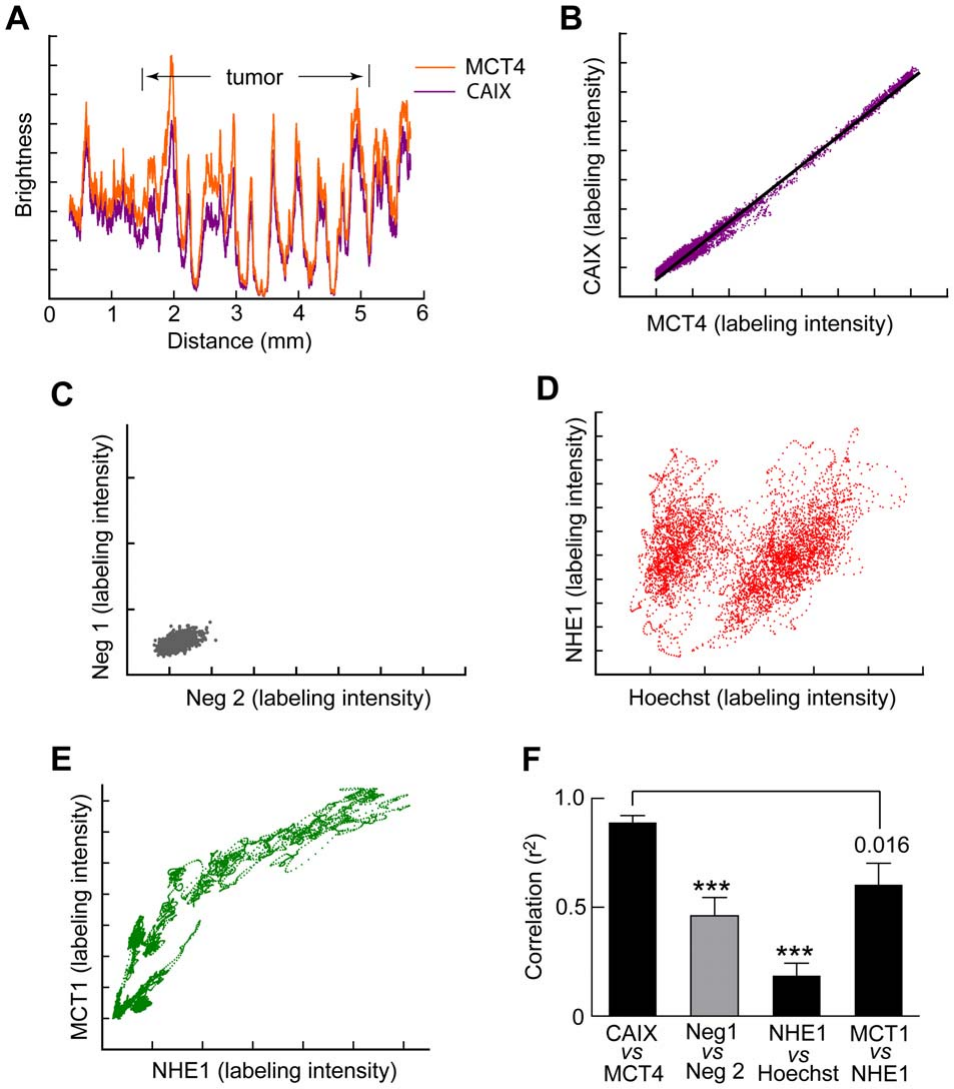
Profiles of CAIX and MCT4 are correlated. (A) Profiles of the intensities of labeling of CAIX and MCT4 along a sample strip 0.3875 mm wide passing through extratumoral tissue and a C6 glioma. The brightness values were scaled to give considerable overlap of the two profiles. (B) The values for CAIX and MCT4 in (A) are highly correlated. (C) The intensity profiles of the secondary antibodies in the absence of primaries show less correlation (ordinate, green fluorescent secondary; abscissa, red fluorescent secondary). (D) NHE1 labeling correlates only weakly with Hoechst labeling. (E). There is moderate correlation of MCT1 with NHE1. (A,B,D,E) are from the same glioma. (F) Average values of r^2^. First column from 8 glioma-bearing brains; other columns from 4,6 and 7 brains respectively (sections labeled for the appropriate pairs were not made for all brains).

Profiles of labeling intensity for MCT4 and CAIX, unlike NHE1 and MCT1, showed no clear organization at the rim of the tumor (Fig. 2 C,E), nor did they show significant correlation on a pixel by pixel comparison (pixel size 0.76 – 1.5 µm). However, on the larger scale of the 0.3875 mm wide bands, the intensity of the two tended to vary in parallel, both outside and inside the tumors, as illustrated in Fig. 3A. From the data that gave Fig. 3A, and other similar profiles, we plotted the intensity of CAIX for each data point against the corresponding intensity of MCT4 at the same spatial position. The points lay close to a straight line characterized by a correlation coefficient r^2^ close to one (Fig. 3 B,F). Part of this correlation is simply due to irregularities in the structure of the tissue in the sections. However, the correlation between the intensities of the unspecific labeling by the pairs of secondary antibodies in the absence of primary antibodies was significantly lower (Fig. 3 C,F). And correlation between Hoechst labeling and each of the proteins was low, even in the case of anti-NHE1 labeling and Hoechst labeling, which peak at about the same distance from the edge (Fig. 3 D,F). The relation between MCT1 and NHE1 may have reflected differences between the intra- and extratumoral compartments (Fig. 3 E); when the average Pearson correlation coefficient was calculated, it was significantly less than for the pair CAIX-MCT4 (Fig. 3F). Mean correlation coefficients were calculated for profiles from each tumor with the appropriate labeling and the averages for the tumors were calculated (Fig. 3F). The mean correlation for CAIX/MCT4 was significantly higher than for the other pairs of labels.

## Discussion

### The tumor rim is structured

The present results show that certain proteins are expressed in an organized way in the rim of a C6 glioma that confronts non-tumoral tissue of the brain parenchyma. Expression of the Na^+^/H^+^ exchanger, NHE1, peaks at about 0.3 mm in from the edge, MCT1 has a broader peak with a maximum at about 1 mm, and, in contrast, CAIX and MCT4 are not significantly upregulated in the rim (Fig. 2). The organized distribution of NHE1 and MCT1 prompts consideration of how these proteins might be involved in fluxes of lactate and protons within tumors. Protons can be transported through tissue in many ways. In extracellular and intracellular fluid, protons can be effectively transported as H_3_O^+^ ions, or on diffusible pH buffer molecules [65]. Movement of CO_2_, or, in the reverse direction, of HCO_3_^−^, is also tantamount to a flux of protons. Effective proton transport across membranes can be mediated by ion channels and H^+^ transporters (such as NHE1 and the MCTs) and also by passage of CO_2_, or, in the reverse direction, by HCO_3_^−^. For brevity, we will generally use “proton transport” to cover all these processes.

### NHE1 expression is upregulated near the edge of the tumor

In “invadopodia” of cells in culture, NHE1 molecules interact with intracellular and extracellular structural proteins to contribute to cell migration. NHE1 also increases pHi and, provided there is a source of intracellular protons to be extruded, decreases pHe, leading to remodeling of the cytoskeleton, dismantling of the extracellular matrix, and cell migration and division [66]. Our observation that expression of NHE1, together with labeling of nucleic acids, peaks near the edge of the C6 glioma, would appear to be compatible with NHE1 having these functions *in vivo*.

The factor by which the number of NHE1 molecules per unit area of membrane is increased in the rim of a C6 glioma is difficult to estimate. The long, fine, processes that greatly increase the membrane areas of neurons and astrocytes, are much reduced in glioma cells. Because of this difference, a given number of NHE1 molecules per unit volume of tissue would correspond to a higher number per unit area of membrane in the glioma than outside it. A contrary tendency arises from the smaller volumes of the glioma cells, particularly in the rim, as evidenced by the greater density of nuclei (e.g., Figs 1 F,G, 2A). However, whatever the area of membrane, an increased number of NHE1 molecules per unit volume of tissue should allow increased extrusion of protons per unit volume. *In vitro*, NHE1 expression can be upregulated by BAX inhibitor 1 [67] and its activity is increased by epidermal growth factor [3], [48].

### The role of MCT1

Extreme hypoxia prevents the oxidation of pyruvate in mitochondria and most of the pyruvate produced by glycolysis is then converted to lactate. Cancer cells in culture, and tumors as a whole, also produce much lactate even when oxygen is available [29], [30], [68]. In exercising striated muscle, it is well established that lactate, exported from white fibers (with predominantly glycolytic metabolism), is taken up on the MCT1 transporter by red fibers with a predominantly oxidative metabolism [32]. Sonveaux *et al* [14] found that MCT1 is upregulated in the rim of SiHa tumors, and that SiHa cells in culture show considerable oxidative metabolism. They suggest that lactate released by cells in hypoxic regions of SiHa tumors is taken up into cells near the edge of the tumor, which, being relatively close to the blood vessels in normal tissue, may receive enough oxygen to oxidize lactate. The peak in MCT1 in the rim of C6 gliomas would allow a similar transfer of lactate from hypoxic to oxygenated regions. Tumors showing this arrangement might prosper. Glucose, about 5 mM in blood, is more plentiful than oxygen, which has a concentration of no more than 2.5 mM in blood (mainly in hemoglobin) and much less in tissue. More oxygen than glucose is required for oxidation of glucose (6 molecules of oxygen per glucose) and, for a parasitic tumor, glucose has no “cost”, while adequate oxygenation deep into a tumor requires the growth of a well-perfused neo-vasculature. Hence, tumors that consume oxygen and lactate in the rim and allow glucose to diffuse deeper into the tumor might grow more rapidly than those that lack this arrangement.

### Creation of an acidic microenvironment

**Figure 4:**
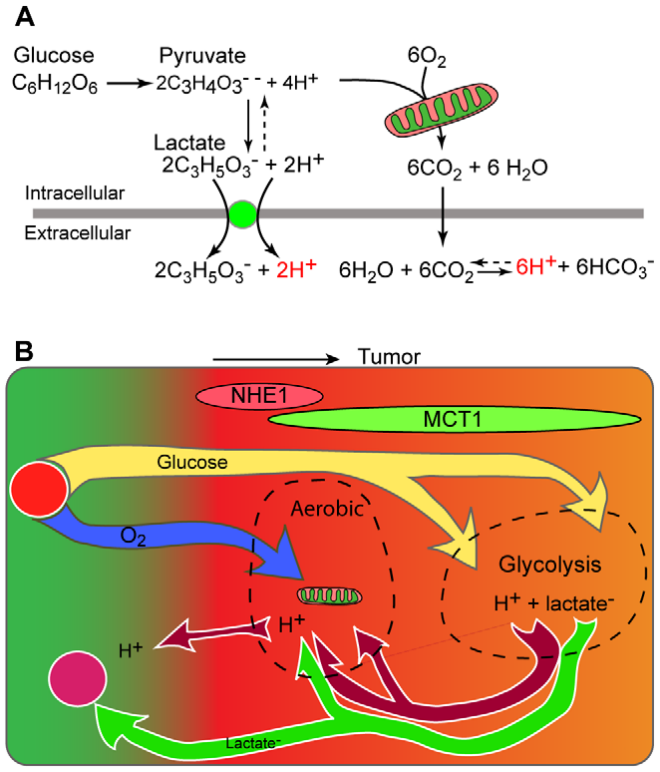
Roles of MCT1 and NHE1 in C6 gliomas. (A) Stoichiometry of the main pathways of glucose metabolism. Note that the protons associated with lactate production are exported by cotransport with lactate. The consequences of CO_2_ production are potentially more variable than shown. (B) Scheme of fluxes of glucose, oxygen, lactate and H^+^ in the glioma rim, suggested by the profiles of NHE1 and MCT1. Some of the lactate exported from deeper, hypoxic regions is taken up on MCT1 in the rim of the tumor and oxidized to CO_2_. Ground colors indicate pHe: red is acid, green is alkaline.

Where do the protons extruded by NHE1 in the rim of the tumor come from? Since an extracellular accumulation of H^+^ will dissipate by diffusion of H^+^ through extracellular space [10], [28], a tumor, as a whole, can only maintain an acidic pHe by constantly extruding protons (or some equivalent process, such as constantly taking up HCO_3_^−^). Any cyclic process whereby protons enter the cell from the extracellular space and are then extruded at almost the same place would not change pHe: the protons must be generated within the cell (or come from other cells – see below). A major source of intracellular H^+^ ions is glycolytic production of lactate. Lactic acid and its precursor, pyruvic acid, are >99% unprotonated at physiological pHs [69]; the stoichiometry of glycolysis for these unprotonated forms is shown in Fig. 4A and it is seen that, in the steady state (when the concentrations of NADH, NAD^+^, ADP, ATP etc. remain constant), lactate ions and H^+^ ions are produced in equal numbers. Extracellular lactate diffuses away through extracellular clefts, so an outwardly directed gradient of [lactate^−^] is created which drives the equimolar cotransport of H^+^ [31]. Since each lactate^−^ crosses the cell membrane in association with a proton, glycolysis does not subject the cell to continuous loading with internally-generated protons, and is not a direct source of protons for other transporters such as NHE1.

Complete oxidation of glucose produces CO_2_ and H_2_O. Normally the net flux of CO_2_ is exported by diffusion, and does not lead to production of intracellular H^+^ (Fig. 4A). In principle, CO_2_ and H_2_O can react within the cell to produce bicarbonate and H^+^ ions and if the bicarbonate is exported, this is a potential source of intracellular protons [5]. However, in the extracellular space, bicarbonate will recombine with H^+^, thereby tending to increase pHe and negate the effects of proton extrusion. Hence, to a first approximation, oxidative phosphorylation is not a useful source of intracellular protons for producing an acid pHe. Synthesis of the components of growing cells, such as fatty acids and proteins, will, in general, be associated with net incorporation of protons [69]. We have not found measurements relating to this, but an extreme possibility for the stoichiometry of saturated fatty acid synthesis from glucose is: (n+2) C_6_ H_12_O_6_ + 12n H → 6(CH_3_-(CH_2_)_n_-CO_2_H) + 6nH_2_O; i.e., a considerable consumption of protons. Hence, growing and dividing cells, which particularly require a source of protons for extrusion on NHE1, also require protons for anabolism.

### The spatial organization of proton fluxes

Tumors release lactate to the blood stream, even though (at least in the case of human colonic carcinomas) the tumor as a whole fails to extract all the available oxygen from the blood [30]. Transformation of a stem cell into a successful tumor is such a rare event that it is possible for tumor cells to differ from normal cells in ways that are complex and apparently coherent in favoring tumor growth [70], [71]. A consequence beneficial to the tumor of releasing lactate, rather using it all to produce large amounts of ATP in the TCA cycle, or to provide hydrocarbon backbones for the synthesis of macromolecules, is the provision of the associated protons to create the acidic microenvironment that promotes growth. The present results, showing a peak of NHE1 expression in the tumor rim, suggest a refinement. H^+^ ions diffusing through extracellular space from deeper in the tumor, enter cells near the rim on transporters or through ion channels and are then available for extrusion on NHE1. This arrangement would not change the total number of H^+^ ions in the extracellular space, but would create the localized decreases in pHe (and increases in pHi) that are part of the processes of cell extension and division [46], [48]. Since the protons were produced in association with lactate, excess lactate would diffuse out of the tumor. In contrast, if protons are taken up into rim cells by cotransport with lactate and the lactate is oxidized to CO_2_ and H_2_O, then the stoichiometry of the reaction requires that these protons are consumed and unavailable for export on NHE1. We are therefore suggesting a conceptual division of the influx of protons into cells in the rim. Some protons enter in association with lactate on MCTs, particularly MCT1, and are consumed in the oxidation of lactate to CO_2_; others enter by other routes and are extruded on NHE1 (Fig. 4B).

### MCT4 and CAIX

Weak CAIX immunoreactivity was found throughout the brain parenchyma, as has been reported by others [72]. Expression in the glioma rim was variable, and on average, not significantly different from extratumoral tissue [56], [59]. Like CAIX, MCT4 was present throughout the parenchyma, in accordance with its reported presence in astrocytes [52]. As in SiHa and Wdr tumors there was little overall upregulation [14]. Strikingly, there was close correlation in space between MCT4 and CAIX on the large scale of hundreds of microns (Fig. 3B,F), but not on a finer scale of microns. *In vitro*, expression of both CAIX and MCT4 is increased by HIF-1αA common upregulatory mechanism, perhaps acting via HIF-1α, might lead to the observed spatial correlation. A close spatial association between a pair of other isoforms of these proteins, MCT1 and CAII, has been reported [62], [73]. CAXII as well as CAIX is upregulated in tumors, and six other known isoforms are present in the brain [74], so our sampling of only CAIX tells us little about the distribution of total CA activity.

### Implications for cancer therapy

Inhibition of either MCT1 or NHE1 has been shown to slow tumor growth in animal models [14], [50], [51]. In our proposed scheme (Fig. 4B), the cells at the rim of the tumor use NHE1 to create the low pHe that favors their extension, migration and proliferation. MCT1 supports not only outward but also inward fluxes of lactate involved in the supply of the hydrocarbon elements necessary for growth, the ATP necessary to maintain proton extrusion, and the protons themselves. NHE1, MCT1, and associated proteins may be molecular targets worth investigating further.

### Conclusion

The arrangement of NHE1 and MCT1 in the rim of C6 gliomas adds to arguments suggesting that net lactate production is beneficial to a tumor because it allows the creation of appropriate intracellular and extracellular pH microenvironments. We need to know if this is true also for other types of tumor and to study the effects, in real time, of inhibiting these, and other, transporters.

## Materials and Methods

### Ethics statement

All procedures involving animals conformed to European Council Directive 86/609/EEC and the study was approved by the Ethical Committee of the Grenoble-Institut des Neurosciences, agreement ID 004. Facilities for animal housing and procedures were approved by the French Ministry of Agriculture, licence A 38 516 10008 and all experimenters held personal licenses. Tumor size was monitored non-invasively by MRI and the rats were sacrificed before the appearance of marked clinical symptoms.

### Preparation of the tumor model

C6 cells [75] from the American Type Culture Collection were grown in DMEM containing 25 mM glucose and 2 mM L-glutamine (product 31966-021 from Invitrogen, Cergy Pontoise, France) to which was added 10% FBS (Invitrogen) and antibiotics. The rat glioma model was prepared as described [76]: male Wistar rats (200–230 g) were anesthetized with isoflurane and 10^5^ C6 cells in DMEM were injected stereotaxically in the right caudate nucleus. About 20–25 days after the tumor implantation, the rat was decapitated, the brain was rapidly removed and frozen in isopentane at – 80°C, and 10 µm cryosections were cut at −20°C.

### Antibodies

In agreement with the vendor's data sheet, we found that the monoclonal anti-NHE1 antibody clone 4E9 (Chemicon MAB3140) did not clearly label sections of brain. In contrast, strong labeling was observed with antiserum 1950 raised against a fusion protein of the C-terminal of human NHE1 (pMAL/NHE1/635-815). This had previously used on pancreatic and kidney tissue [77] and we further demonstrated its specificity (see Supporting Information, Text S1, Figure S2).

For MCT1 we used VPA 1286 from Abcys, Paris, at 1/300. This is a chicken polyclonal antibody raised against a 25 AA peptide from the cytoplasmic C terminal of rat MCT1. Affinity purified rabbit anti MCT4 was MCT45-A from Alpha Diagnostic, San Antonio, used at 1/300. The monoclonal antibody M25 against carbonic anhydrase IX (CAIX) has been described [72]. Secondary antibodies were anti rabbit Alexa 568, anti-mouse Alexa 488 or anti-chicken Alexa 488, all at 1/500 and from Invitrogen.

### Immunohistochemistry

The sections were fixed for 10 min in 4% paraformaldehyde, washed and incubated for 1 h in 3% BSA at room temperature, then incubated with the first antibody in 3% BSA for 16 h at 4°C. After 3 rinses in PBS, the secondary antibody was applied for 1 h at room temperature. After three more rinses, the sections were mounted in GelMount (MM, France) containing bisbenzimide Hoechst 33342 trichlorohydride.

### Imaging

Tile scans were made on a Leica DM6000B microscope with a TCS SP5 confocal system. For profiles, a x40 oil immersion objective (Leica HCX PL APO) was used with the numerical aperture reduced to 0.75 to provide uniform illumination of the field. The pinhole was set at 200 µm to increase the depth of field and reduce errors due to changes in the plane of the section, and each frame was 512×512 pixels. Analysis was done with ImageJ and GraphPad Prism. To average profiles, the individual raw profiles were smoothed twice over 13 points and the edge of the tumor defined as x = 0 on a graph of the Hoechst labeling. The abscissae for the profiles of the other labels (e.g., NHE1 and MCT1) were shifted by the same amount. The profiles from all the tumors were averaged, the S.E.M. for each x value being calculated by Prism.

## Supporting Information

Figure S1 **Immunolabeling of tissue sections.** (A) NHE1 labeling outside a tumor. (B) Negative control (no primary antibody) on an adjacent section. (C) Higher magnification of double labeling for NHE1 (red) and MCT1 (green) showing MCT1 labeling along blood vessels in extratumoral tissue.
(TIF)

Figure S2 **Specificity of the anti-NHE1 antiserum.** (A) Western blot of antiserum 1950 on a protein extract of a brain bearing a C6 glioma showing bands at 95 and 65 kDa. (B) The preparation of CterNHE1-GST revealed with Coomassie blue in SDS-PAGE on a 10% gel (a) and revealed on Western blots by antiserum 1950 (b) and monoclonal antibody 4E9 (c). After depletion by the NHE1 construct, antiserum 1950 no longer detected the construct (d). Labeling of brain sections was more intense with undepleted antiserum 1950 (C) than with the depleted antiserum (D). Antiserum 1950 did not label the NHE3 isoform in PS 120 fibroblasts (E). WT fibroblasts transfected to express NHE1 show a band at the appropriate MWt (arrow), whereas this is not present for WT fibroblasts and fibroblasts transfected to express NHE3. Scale bars in (C,D) 100 mm.
(TIF)

Text S1 **Tests showing the efficacy of the anti-NHE1 antiserum.**
(DOC)
