# Supplementary figures and images for: The Spatial Organization of Proton and Lactate Transport in a Rat Brain Tumor
Source: PLoS One. 2011 Feb 24;6(2):e17416. doi: 10.1371/journal.pone.0017416 (PMC3044751; doi:10.1371/journal.pone.0017416)

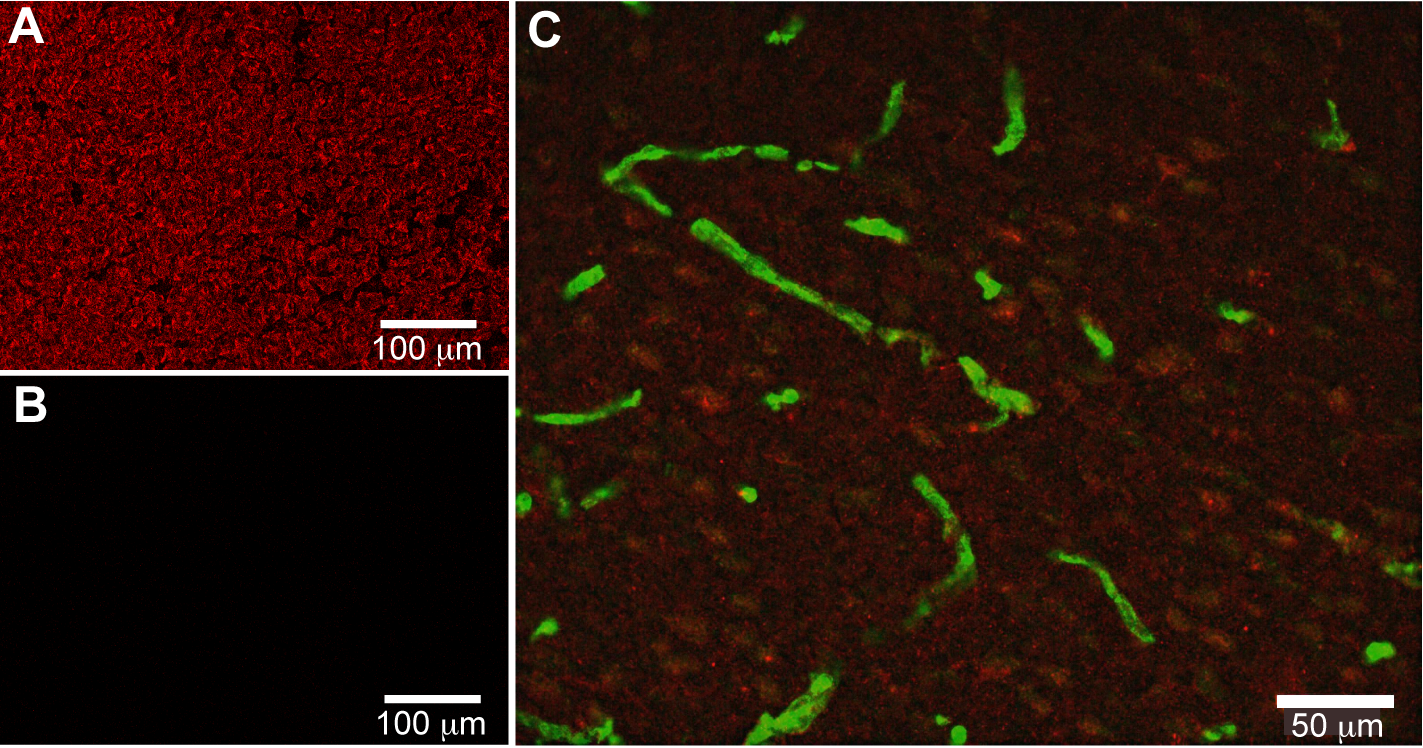

Supplement: Figure S1 — Immunolabeling of tissue sections. (A) NHE1 labeling outside a tumor. (B) Negative control (no primary antibody) on an adjacent section. (C) Higher magnification of double labeling for NHE1 (red) and MCT1 (green) showing MCT1 labeling along blood vessels in extratumoral tissue. (TIF) [file pone.0017416.s001.tif]

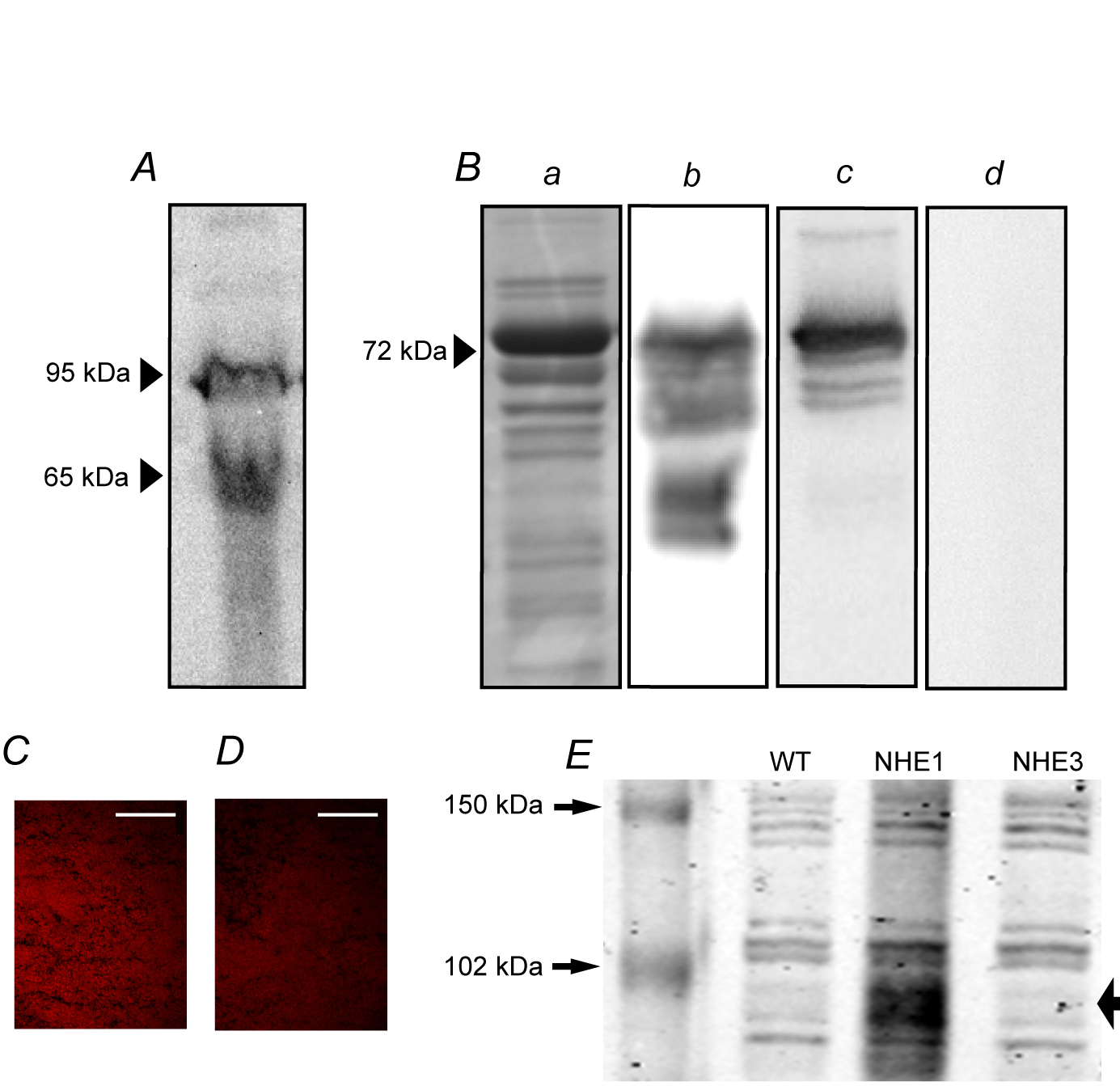

Supplement: Figure S2 — Specificity of the anti-NHE1 antiserum. (A) Western blot of antiserum 1950 on a protein extract of a brain bearing a C6 glioma showing bands at 95 and 65 kDa. (B) The preparation of CterNHE1-GST revealed with Coomassie blue in SDS-PAGE on a 10% gel (a) and revealed on Western blots by antiserum 1950 (b) and monoclonal antibody 4E9 (c). After depletion by the NHE1 construct, antiserum 1950 no longer detected the construct (d). Labeling of brain sections was more intense with undepleted antiserum 1950 (C) than with the depleted antiserum (D). Antiserum 1950 did not label the NHE3 isoform in PS 120 fibroblasts (E). WT fibroblasts transfected to express NHE1 show a band at the appropriate MWt (arrow), whereas this is not present for WT fibroblasts and fibroblasts transfected to express NHE3. Scale bars in (C,D) 100 mm. (TIF) [file pone.0017416.s002.tif]
