# Supplementary material for: The Spatial Organization of Proton and Lactate Transport in a Rat Brain Tumor
Source: PLoS One. 2011 Feb 24;6(2):e17416. doi: 10.1371/journal.pone.0017416 (PMC3044751; doi:10.1371/journal.pone.0017416)
Supplement: Text S1 — Tests showing the efficacy of the anti-NHE1 antiserum. (DOC) [file pone.0017416.s003.doc]

**Non-specific labeling by the secondary antibodies**

At high magnification, the immunolabeling showed cellular structure, as in Figure S1C. Since we are interested in much larger scales, we obtained relatively smooth profiles by imaging broad (387.5 μm) strips of tissue. Large polygonal regions of interest (ROIs) were drawn over tumor tissue and extratumoral tissue and labeling intensities measured (in arbitrary units) on sections with both primary and secondary antibodies and on neighboring sections without primary antibodies (Figure S1A,B). The ratios with/without primary antibody were very variable, ranging from 6 to 36, so intensity profiles were not corrected for non-specific labeling by the secondary antibodies. This omission causes slight underestimation of the true ratios of peak intensity to baseline. For example, if the ratio without/with primary were 0.1, the true value of the ratio NHE1 (peak in tumor)/(extratumoral tissue) would be 1.60 rather than the uncorrected value of 1.55.

**The specificity of the anti-NHE1 antiserum.**.

On Western blots of a protein extract from a glioma-bearing brain, it labeled bands corresponding to MWts of 95 and 65 kDa (Figure S2 A). These correspond approximately with the values of 110 and 85 kDa reported by Noël *et al*.[1] for MDCK cells, and 100 and 65 kDa reported by Moulin *et al*. [2] for rat pancreas and kidney. A second CterNHE1-GST construct was prepared independently and partially purified (Figure S2Ba). On this preparation, the main protein recognized by antiserum 1950 had the same MWt as that recognized by the commercial monclonal antibody clone 4E9 (MAB3140, Chemicon; Fig. S2 Bb,c). Antiserum 1950 that was depleted by the CterNHE1-GST construct did not detect the construct (Fig. S1 Bd); the depleted antiserum gave weaker labeling on brain sections (Figure S2 C,D). Antiserum 1950 did not cross-react with NHE3 (Figure S2 E)

1. Noel J, Roux D, Pouyssegur J (1996) Differential localization of Na^+^/H^+^ exchanger isoforms (NHE1 and NHE3) in polarized epithelial cell lines. J. Cell Sci. 109:929-939.

2. Moulin P, Guiot Y, Jonas JC, Rahier J, Devuyst O*, et al.* (2007) Identification and subcellular localization of the Na^+^/H^+^ exchanger and a novel related protein in the endocrine pancreas and adrenal medulla. J. Mol. Endocrinol. 38:409-422.

**Figure S1. Immunolabeling of tissue sections.** (A) NHE1 labeling outside a tumor. (B) Negative control (no primary antibody) on an adjacent section. (C) Higher magnification of double labeling for NHE1 (red) and MCT1 (green) showing MCT1 labeling along blood vessels in extratumoral tissue.

**Figure S2. Specificity of the anti-NHE1 antiserum.** (A) Western blot of antiserum 1950 on a protein extract of a brain bearing a C6 glioma showing bands at 95 and 65 kDa. (B) The preparation of CterNHE1-GST revealed with Coomassie blue in SDS-PAGE on a 10 % gel (a) and revealed on Western blots by antiserum 1950 (b) and monoclonal antibody 4E9 (c). After depletion by the NHE1 construct, antiserum 1950 no longer detected the construct (d). Labeling of brain sections was more intense with undepleted antiserum 1950 (C) than with the depleted antiserum (D). Antiserum 1950 did not label the NHE3 isoform in PS 120 fibroblasts (E). WT fibroblasts transfected to express NHE1 show a band at the appropriate MWt (arrow), whereas this is not present for WT fibroblasts and fibroblasts transfected to express NHE3. Scale bars in (C,D) 100 μm.
